# Bioremediation of Parboiled Rice Effluent Supplemented with Biodiesel-Derived Glycerol Using *Pichia pastoris* X-33

**DOI:** 10.1100/2012/492925

**Published:** 2012-07-31

**Authors:** Diego Gil de los Santos, Carlos Gil Turnes, Fabricio Rochedo Conceição

**Affiliations:** ^1^Núcleo de Biotecnologia, Centro de Desenvolvimento Tecnológico, Universidade Federal de Pelotas, 96010-900 Pelotas, RS, Brazil; ^2^Instituto Federal de Educação, Ciência e Tecnologia Sul-rio-grandense, 96015-360 Pelotas, RS, Brazil

## Abstract

This paper describes the use of *Pichia pastoris* X-33 as a bioremediator to reduce the chemical oxygen demand (COD), total Kjeldahl nitrogen (TKN), and phosphorus (P-PO_4_^   3−^), after culture in parboiled rice effluent supplemented with p.a. glycerol or a glycerol by-product of the biodiesel industry. The greatest reduction in the COD (55%), TKN (45%), and P-PO_4_^   3−^ (52%) of the effluent was observed in cultures of *P. pastoris* X-33 supplemented with 15 g *·*L^−1^ of biodiesel-derived glycerol. Furthermore, the overall biomass yield was 2.1 g *·*L^−1^. These data suggest that biodiesel-derived glycerol is an efficient carbon source for the bioremediation of parboiled rice effluent and biomass production.

## 1. Introduction

Brazil is the ninth largest producer of rice in the world, 12,600 thousand tonnes in 2009, 20% of which was parboiled. The parboiling process produces an estimated two litres of effluent per kilo of rice, equivalent to 504 billion litres of effluent per year in Brazil alone. Parboiled rice effluent contains high levels of organic matter, expressed as the chemical oxygen demand (COD), as well as significant levels of nitrogen and phosphorus that can be used in the production of single cell protein (SCP) for use in animal feed [1]. The application of yeast to the treatment of liquid waste has been in use since the seventies, to reduce the COD, the biological oxygen demand (BOD), and nitrogen, thereby decreasing the environmental impact as well as producing SCP for use in animal feed [2–4]. Recently, yeast strains were selected to enhance phosphorus removal in industrial effluent [5]. However, the use of *Candida utilis* to reduce nitrogen levels in parboiled rice effluent was not encouraging [3]. Nevertheless, it has been possible to growth of *Saccharomyces boulardii* and *Pichia pastoris* in effluent supplemented with an additional carbon source [6].

Research into alternative renewable energy sources has stimulated the development and promotion of the biofuel industry. Brazil is the world's third largest biodiesel producer and generates over 300 tonnes of by-product during the transesterification of vegetable oils. This by-product contains glycerol, methanol, volatile acids, and other elements. This by-product has a low commercial value when it is not purified to pharmacological grade and is a major challenge to the biodiesel industry due to the huge volumes that must be processed. Recently, there have been reports of microorganisms capable of converting the glycerol by-product into value-added products [7].

*Pichia pastoris* is a methylotrophic yeast mainly used for heterologous protein expression and that can yield high cell concentrations when glycerol is available [8]. It has a generally recognized-as-safe (GRAS) status as well as probiotic properties [9, 10]. To reduce the environmental impact of parboiled rice effluent it can be supplemented with biodiesel-derived glycerol, and the resulting yeast culture may be used as probiotic. However, there is no information on its use in the bioremediation of parboiled rice effluent. Therefore, the objectives of this study were to evaluate the growth of *P. pastoris *X-33 in parboiled rice effluent supplemented with a biodiesel by-product (containing glycerol) and determine its impact on the environmental parameters (COD, TKN and phosphorous) of the effluent and on biomass production.

## 2. Materials and Methods

### 2.1. Strain and Culture Conditions

*Pichia pastoris* X-33 (Invitrogen, USA) was grown in YM broth (Yeast Medium, Difco, USA) at 28°C for 12 h at 150 rpm in an orbital shaker and was used to inoculate 500 mL baffled flasks containing 20% (volume) of the glycerol supplemented effluent. Five media were tested: parboiled rice effluent without supplement (AP); supplemented with 5 (AP/G5) or 15 (AP/G15) g·L^−1^ of p.a. glycerol; supplemented with 5 (AP/B5) and 15 (AP/B15) g·L^−1^ of crude soybean biodiesel by-product (biodiesel glycerol). The pH was adjusted to 5.5 with 1 N NaOH, and the media was sterilized at 121°C for 15 minutes.

### 2.2. Parboiled Rice Effluent

The parboiled rice effluent was obtained from the maceration tanks of a parboiled rice manufacturer located in the State of Rio Grande do Sul, Brazil, and was collected at four different months during 2010. The samples were collected in sterile containers, autoclaved at 121°C for 15 min and maintained under refrigeration until used.

### 2.3. Glycerol

Crude glycerol a by-product of the soybean biodiesel industry (biodiesel glycerol) and p.a. glycerol (*Synth*, Brazil) were used as supplements in the parboiled rice effluent. The characteristics of the biodiesel glycerol are shown in Table 1.

### 2.4. Analytical Methods

 Samples were collected 0 and 70 h after inoculation of the respective media, the pH was adjusted to 2.0 with H_2_SO_4_, and the samples were stored at 4°C until further use. The samples were centrifuged at 1800 *g* for 10 min, and the supernatant was used to determine the COD, TKN, and phosphorous (P-PO_4_^3−^) using standard, previously described methods [11]. Briefly, the pellets were washed three times in sterile water and dried at 80°C to a constant weight. The COD was determined by open reflux testing, total nitrogen by the Kjeldahl method and phosphorus by subjecting the samples to sulphuric-nitric acid digestion and then measuring the inorganic phosphate (P-PO_4_^3−^) level by the ascorbic acid method. The chemical analyses were repeated and resulted in variation of less than 10% (data not shown).

### 2.5. Statistical Analysis

The data were analyzed by ANOVA, and differences among the means were compared using the Tukey test at 5% significance level using Statistics software version 7 (Statsoft, USA). The COD, TKN, and P-PO_4_^−3^ results were evaluated for normality using the Shapiro-Wilk test.

## 3. Results and Discussion

The effluent produced by rice parboiling contains nutrients that can be used by bacteria and yeast to produce SCP [1, 6] and in bioremediation to reduce the COD, BOD, and TKN, thereby diminishing the environmental impact of the effluent [2–4, 12].

**Figure 1:**
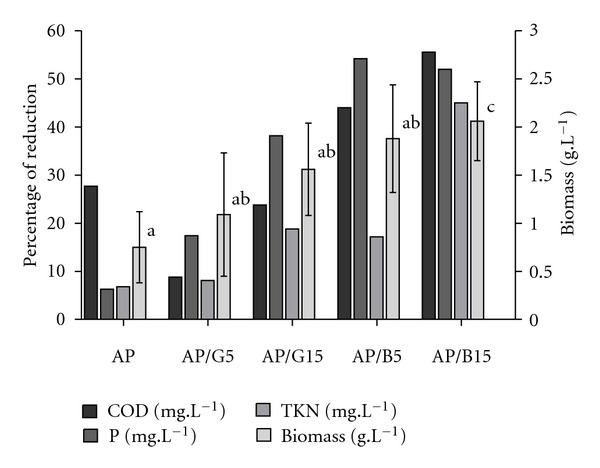
Chemical oxygen demand (COD), phosphorous (P-PO_4_^3−^), nitrogen (TKN), and biomass after 70 h of culture in effluent from parboiled rice industry, with or without p.a. or biodiesel glycerol. AP: plain effluent from rice parboiled maceration tanks; AP/G5 supplemented with 5 g·L^−1^ of p.a. glycerol.; AP/G15 with 15 g·L^−1^ p.a. glycerol; AP/B5 with 5 g·L^−1^ of biodiesel glycerol; AP/B15 with 15 g·L^−1^ of biodiesel glycerol. Different superscripts letters: significant differences (*α* = 0.05).

The experiments demonstrated (Figure 1) that *P. pastoris *X-33, originally developed for the expression of heterologous proteins, can be used to improve environmental parameters of parboiled rice effluent, reducing the COD, TKN, and phosphorus (P-PO_4_^3−^) levels, as well as producing significant amounts of SCP, independent of the effluent composition. Additionally, we demonstrated that biodiesel glycerol, a by-product of the soybean biodiesel industry with no intrinsic commercial value, could be used to improve the SCP or probiotic yield. The authors believe this to be the first report of the application of *P. pastoris* to the bioremediation of parboiled rice effluent.

The dry biomass yield of *P. pastoris* X-33 following 70 h of culture in various effluents is shown in Figure 1. Supplementation with 5 and 15 g·L^−1^ of p.a. glycerol increased biomass by 45 and 100%, respectively, compared to when the yeast was grown in the nonsupplemented effluent. The same concentrations of biodiesel glycerol increased the yield by 150 and 175%, respectively. Of note, the biomass yield obtained with rice effluent supplemented with 15 g·L^−1^ biodiesel glycerol was significantly higher (*P* < 0.05) than the nonsupplemented effluent (AP).

The inherent variation in the composition of the effluents influenced the COD, TKN, and phosphorus values (Table 2), in agreement with previous studies [1, 12]. The smaller reduction in the COD, TKN and phosphorus levels of the nonsupplemented effluents suggested that only a fraction of the carbon (like COD), nitrogen, and phosphorus of the effluent could be assimilated by the yeast and converted into biomass. However, the mean yield of 0.75 g·L^−1^ observed in this study was 60% higher than that obtained with the microalgae *Aphanothece microscopica Nägeli* in a similar effluent [12, 13].

Glycerol is an important substrate for several species of microorganism. There are several protocols for *P. pastoris* using glycerol as the main carbon source in order to increase biomass [8]. Supplementation of the effluent with 5 g·L^−1^ and 15 g·L^−1^ of p.a. glycerol or biodiesel glycerol resulted in increased dry biomass (Figure 1), although only the higher supplement had a significant impact (Figure 1). The highest biomass production was obtained with 15 g·L^−1^ of biodiesel glycerol, achieving a mean of 2.06 g·L^−1^of dry biomass. This value is approximately 20% of those reported by Çelik et al. [14] using recombinant *P. pastoris* grown in a medium containing 12.6 g·L^−1^ of glycerol obtained from canola oil, yeast extract, peptone, YNB, and biotin, a richer and more expensive medium than that used in this study.

The addition of 15 g·L^−1^ of biodiesel glycerol to the effluent increased the dry biomass yield by 175% and significantly reduced (*P* < 0.05) the COD compared to the cultures grown in effluent alone or supplemented with p.a. glycerol. This may be due to the assimilation of carbon from other sources in the biodiesel glycerol. Biodiesel glycerol contains fatty acids, methanol and trace elements [15] that possibly interfere in cellular metabolism, increasing biomass production and consequently reducing the COD, TKN, and phosphorous levels. The reduction in the COD of the effluent supplemented with p.a. glycerol was lower than in the effluent only culture, probably because the yeast could not assimilate all of the added glycerol (Table 2).

 The addition of either biodiesel or p.a. glycerol produced a significant reduction in the phosphorus levels, probably due to the increase in biomass production (Table 2), suggesting that it was metabolized during culture. Phosphorus removed from wastewater by enhanced biological phosphorus removal plant was reported to accumulate in bacteria [16] and in *Saccharomyces cerevisiae *[17] in the form of inorganic phosphate granules (Poli-P). The highest phosphorus removal rates were observed in cultures supplemented with 5 and 15 g·L^−1^ of biodiesel glycerol (54 and 52% respectively). The reduction observed in this study was higher than those obtained in an UASB reactor operating in aerobic and anaerobic cycles (17.8%) [18] and with facultative lagoons or activated sludge (<35%) [19]. UASB is an anaerobic system widely used in the treatment of parboiled rice effluent but it has a poor nitrogen and phosphorus removal capacity, thereby requiring the use of additional downstream treatments such as aerated lagoons, wetlands with a removal efficiency of 70–90% [20], and physicochemical phosphorus precipitation with 95% efficient. However, these systems produce residues with high phosphorus contents. The rates of phosphorus removal by *P. pastoris* X-33 observed in this study in the presence of biodiesel glycerol are encouraging, considering that they were obtained in shaker flasks and that the produced biomass may be used as probiotic.

The TKN removal efficiency of *P. pastoris* in effluent alone was 6.8% (Table 2), lower than those observed with the supplemented effluent. The highest nitrogen removal efficiency (45%) was obtained with the addition of 15 g·L^−1^ of biodiesel glycerol, demonstrating that the increase in biomass improved the removal efficiencies of the COD and phosphorous in the effluent. The nitrogen removal rate is similar to those reported by Rodrigues and Koetz [3] using *C. utilis* and with facultative lagoons or activated sludge as described by Von Sperling and Oliveira [19]. However, the algae *A. microscopica Nägeli* demonstrated the best nitrogen removal efficiency, 73% [12], higher than those obtained by yeast culturing.

The results showed that *Pichia pastoris *X-33 may improve the quality of parboiled rice effluents, lowering the COD, the total nitrogen, and phosphorus concentrations, as well as producing a biomass that could be used as SCP or probiotic.

## 4. Conclusion

*Pichia pastoris* X-33 grown for 70 h in parboiled rice effluents supplemented with 15 g·L^−1^ of biodiesel glycerol yielded 2.06 g·L^−1^ of dry biomass and reduced the COD by 55%, phosphorus by 52%, and total Kjeldahl nitrogen by 45%.

## Figures and Tables

**Table 1:** Composition of the crude soybean biodiesel by-product (biodiesel glycerol).

Parameter	Value	Unit	Method^a^
Volatiles	26.60	% mass	AOCS C_a_ 2c-25
Combined alkalinity	1.80	% mass	AOCS C_c_ 17-95
Methanol	1.37	% mass	EN 14110
Ash	5.30	% mass	ASTM D 874
Specific density	1.30	g·cm^−3^	NBR 7148
Acid index	1.70	% mass	ASTM D 664
Glycerol content	54.74	% mass	AOCS 14-46
COD	1.328.040	mg O_2_·L^−1^	[11]
P-PO_4_^3−^	420	mg P·L^−1^	[11]

^a^Official Methods of Analysis of AOAC; EN: European Standards; ASTM: American Society for Testing and Materials; NBR: Associação Brasileira de Normas Técnicas.

**Table 2:** Removal, chemical oxygen demand (COD), phosphorus (P-PO_4_^3−^), and total Kjeldahl nitrogen (TKN) means in rice parboiled effluents after culture of *P. pastoris* X-33 in four different months.

Treatment	COD (mg·L^−1^)			N-TKN (mg·L^−1^)			P ( mg·L^−1^)		
	0 h	70 h	% reduction compared with control at 70 h	0 h	70 h	% reduction compared with control at 70 h	0 h	70 h	% reduction compared with control at 70 h
AP (control)	6422.7 ± 1648.0	4646.0 ± 1015.4	0.0^b^	229.0 ± 81.6	213.5 ± 53.6	0.0^a^	87.8 ± 44.6	82.3 ± 45.1	0.0^a^
AP/G5	6300.8 ± 2165.3	5747.6 ± 3030.4	−23.7^a^	204.7 ± 66.0	188.1 ± 32.7	11.9^a.b^	67.5 ± 10.0	55.8 ± 29.1	32.2^b^
AP/G15	6674.0 ± 2003.6	5087.4 ± 2318.6	−9.5^b^	230.1 ± 56.2	187.0 ± 61.2	12.4^a.b^	86.5 ± 38.1	53.5 ± 38.7	35.0^c^
AP/B5	7002.6 ± 2563.5	3922.3 ± 1296.3	15.6^c^	187.0 ± 68.8	154.9 ± 35.7	27.5^b.c^	99.1 ± 51.5	45.4 ± 28.7	44.8^d^
AP/B15	8051.9 ± 2110.2	3572.8 ± 954.0	23.1^c^	245.6 ± 48.4	135.0 ± 54.5	36.8^c^	98.1 ± 51.7	47.1 ± 29.9	42.7^d^

AP: plain effluent from rice parboiled maceration tanks; AP/G5 supplemented with 5 g·L^−1^ of p.a. glycerol.; AP/G15 with 15 g·L^−1^ p.a. glycerol; AP/B5 with 5 g·L^−1^ of biodiesel glycerol; AP/B15 with 15 g·L^−1^ of biodiesel glycerol. Different superscripts letters mean significant differences after 70 h of culture (*α* = 0.05).

## References

[1] Queiroz M, Koetz PR (1997). Caracterização do efluente da parboilização do arroz. *Revista Brasileira de Agrociência*.

[2] Chanda S, Chakrabarti S (1996). Plant origin liquid waste: a resource for single cell protein production by yeast. *Bioresource Technology*.

[3] Rodrigues R, Koetz P (1996). Remoção de nitrogênio de efluente da indústria de arroz parboilizado por incorporação em biomassa celular de *Candida utilis*-IZ-1840. *Revista Brasileira de Agrociência*.

[4] Choi MH, Park YH (2003). Production of yeast biomass using waste Chinese cabbage. *Biomass and Bioenergy*.

[5] Watanabe T, Masaki K, Iwashita K, Fujii T, Iefuji H (2009). Treatment and phosphorus removal from high-concentration organic wastewater by the yeast *Hansenula anomala* J224 PAWA. *Bioresource Technology*.

[6] Schneid AS, Gil de los Santos JR, Elías M, Gil-Turnes C Wastewater of rice parboiling process as substrate for probiotics.

[7] Chatzifragkou A, Makri A, Belka A (2011). Biotechnological conversions of biodiesel derived waste glycerol by yeast and fungal species. *Energy*.

[8] Higgings DR, Cregg M (1998). *Methods in Molecular Biology – Pichia Protocols*.

[9] Storch OB, Conceição FR, Gil de los Santos JR, Gil-Turnes C *Pichia pastoris* increased humoral response and feed efficiency in broilers.

[10] Gil de los Santos JR, Storch OB, Fernandes CG, Gil-Turnes C (2012). Evaluation in broilers of the probiotic properties of Pichia pastoris and a recombinant *Pichia pastoris* containing the Clostridium perfringens alpha toxin gene. *Veterinary Microbiology*.

[11] APHA (1998). *Standard Methods for the Examination of Water and Wastewater*.

[12] Queiroz MI, Jacob-Lopes E, Zepka LQ, Bastos RG, Goldbeck R (2007). The kinetics of the removal of nitrogen and organic matter from parboiled rice effluent by cyanobacteria in a stirred batch reactor. *Bioresource Technology*.

[13] Zepka LQ, Jacob-Lopes E, Goldbeck R, Queiroz MI (2008). Production and biochemical profile of the microalgae *Aphanothece microscopica Nägeli* submitted to different drying conditions. *Chemical Engineering and Processing*.

[14] Çelik E, Ozbay N, Oktar N, Çalik P (2008). Use of biodiesel byproduct crude glycerol as the carbon source for fermentation processes by recombinant *Pichia pastoris*. *Industrial and Engineering Chemistry Research*.

[15] Thompson JC, He BB (2006). Characterization of crude glycerol from biodiesel production from multiple feedstocks. *Applied Engineering in Agriculture*.

[16] Gebremariam SY, Beutel MW, Christian D, Hess TF (2011). Research advances and challenges in the microbiology of enhanced biological phosphorus removal—a critical review. *Water Environment Research*.

[17] Vagabov VM, Trilisenko LV, Kulakovskaya TV, Kulaev IS (2008). Effect of a carbon source on polyphosphate accumulation in *Saccharomyces cerevisiae*. *FEMS Yeast Research*.

[18] Faria OLV, Koetz PR, Dos Santos MS, Nunes WA (2006). Rice parboilization wastewater phosphorus removal by enhanced biological assimilation in sequencing batch reactor (SBR). *Ciencia e Tecnologia de Alimentos*.

[19] Von Sperling M, Oliveira SC (2009). Comparative performance evaluation of full-scale anaerobic and aerobic wastewater treatment processes in Brazil. *Water Science and Technology*.

[20] Gerber M (2002). *Tratabilidade de efluentes da parboilização de arroz em sistema com plantas aquáticas emergentes*.

